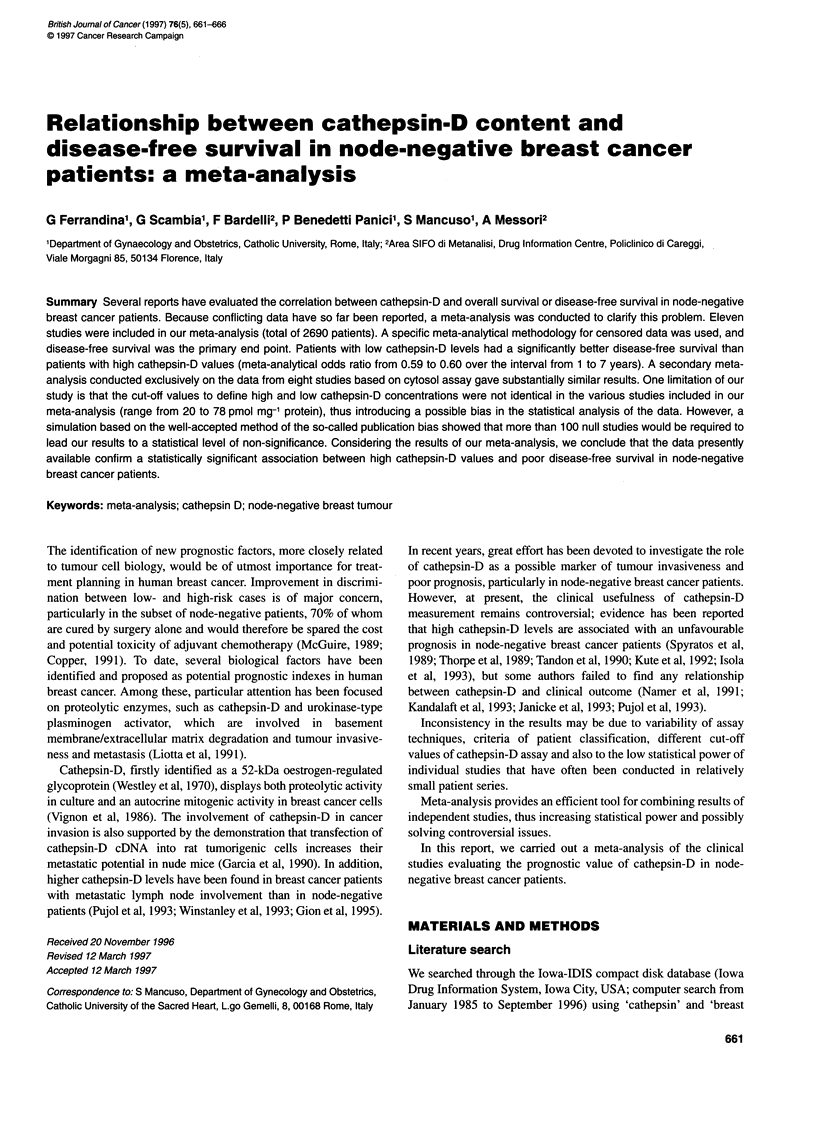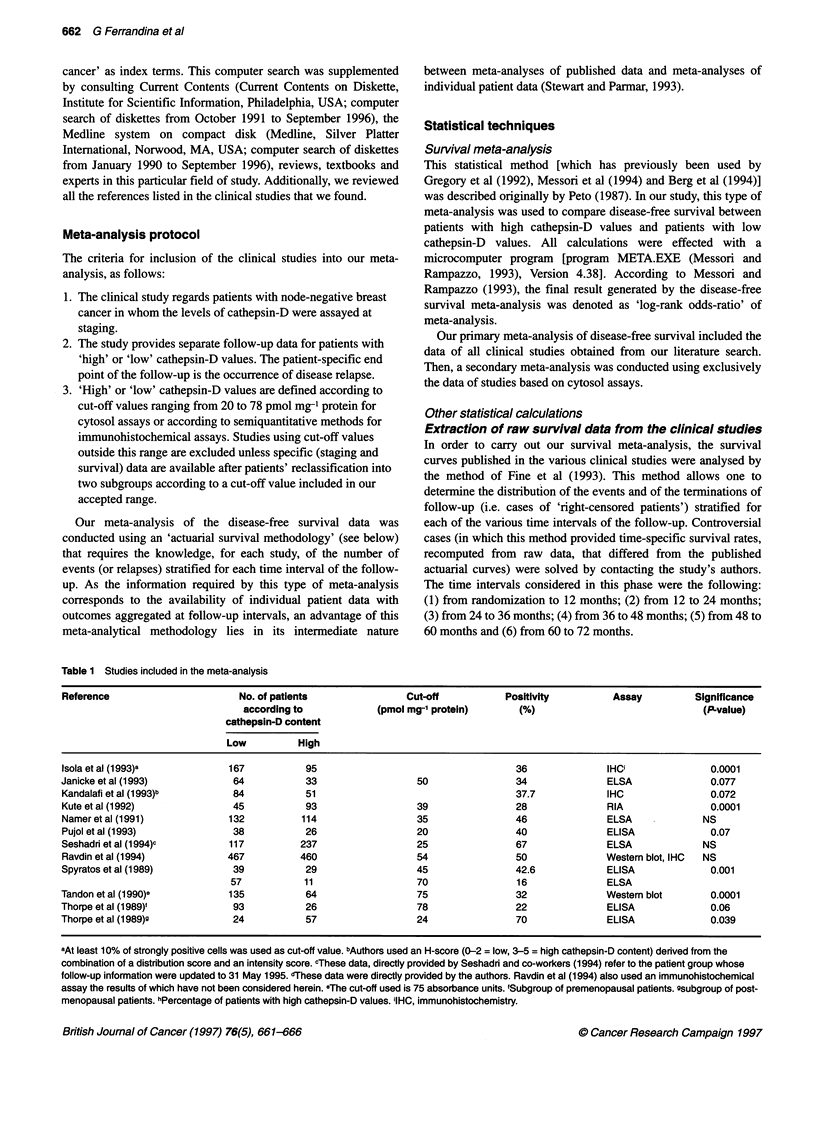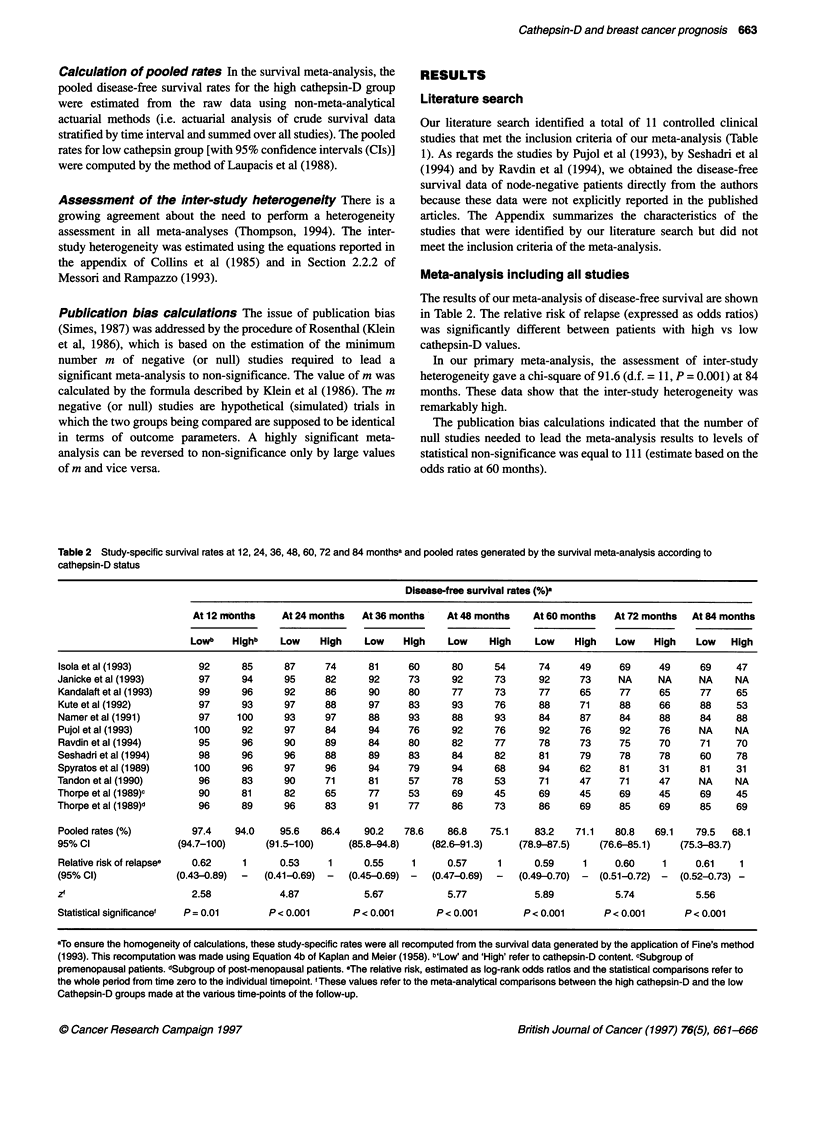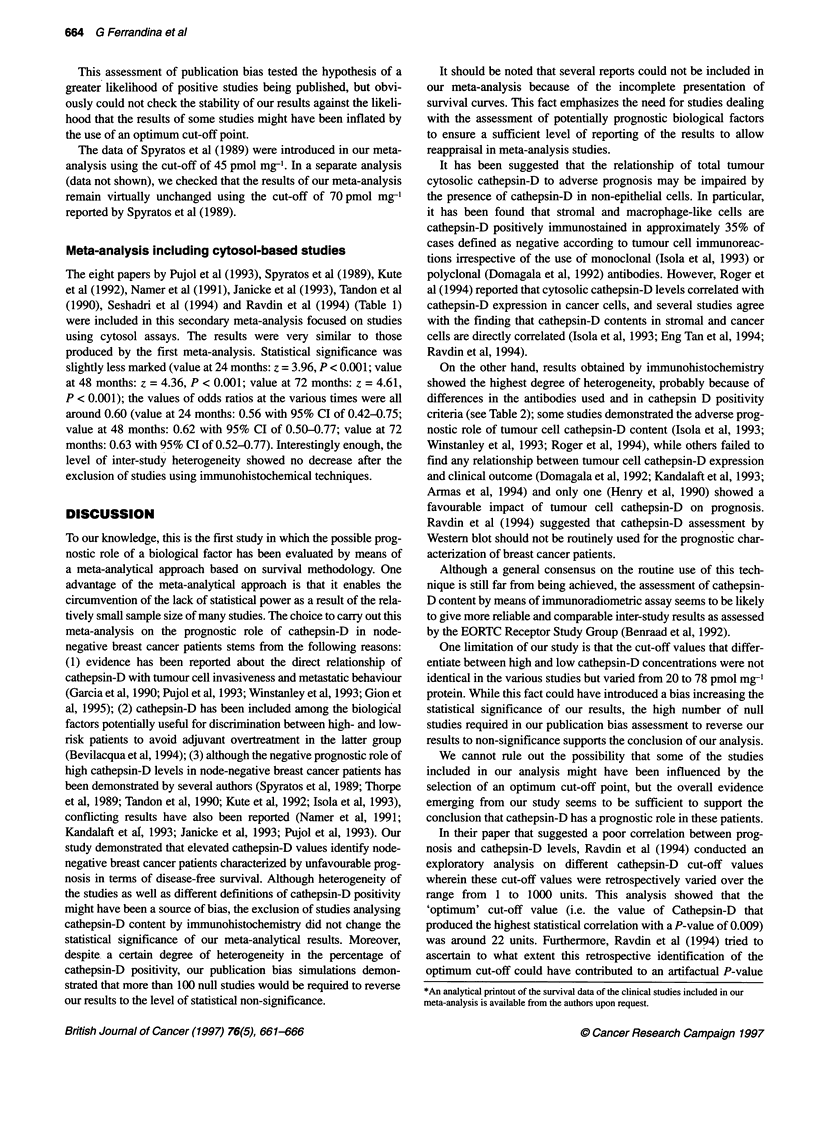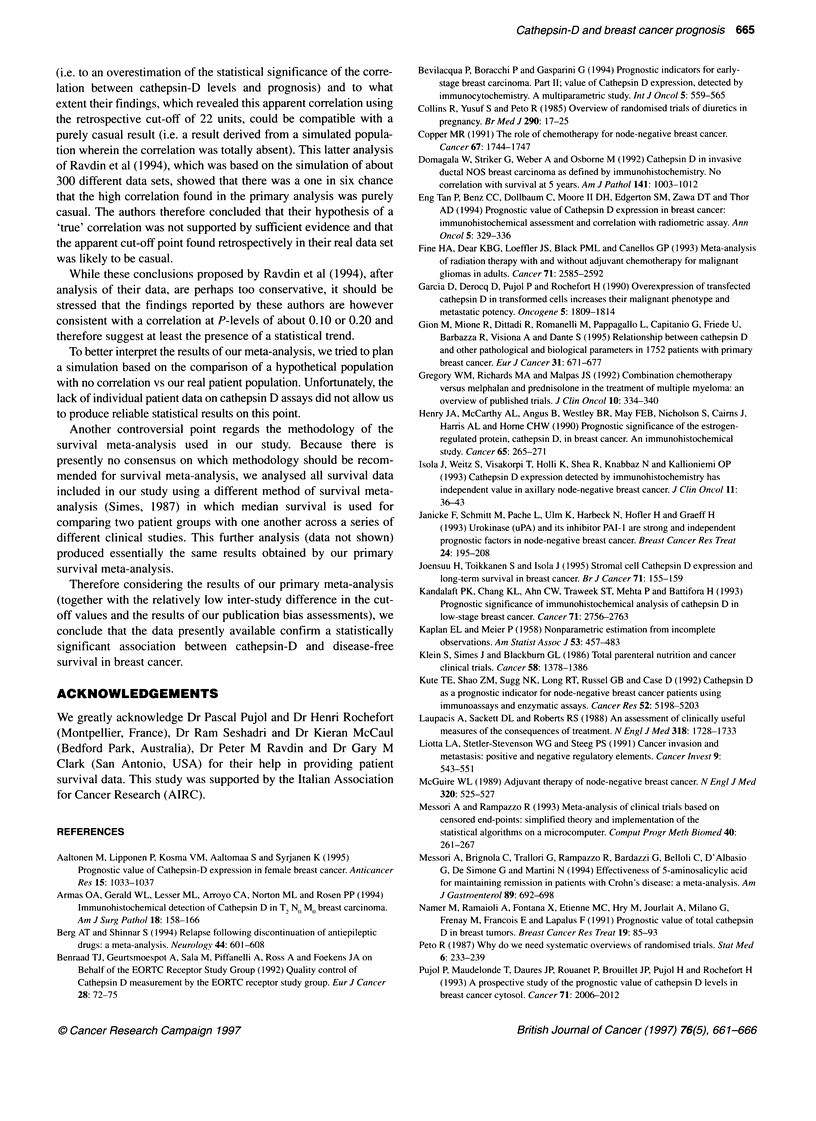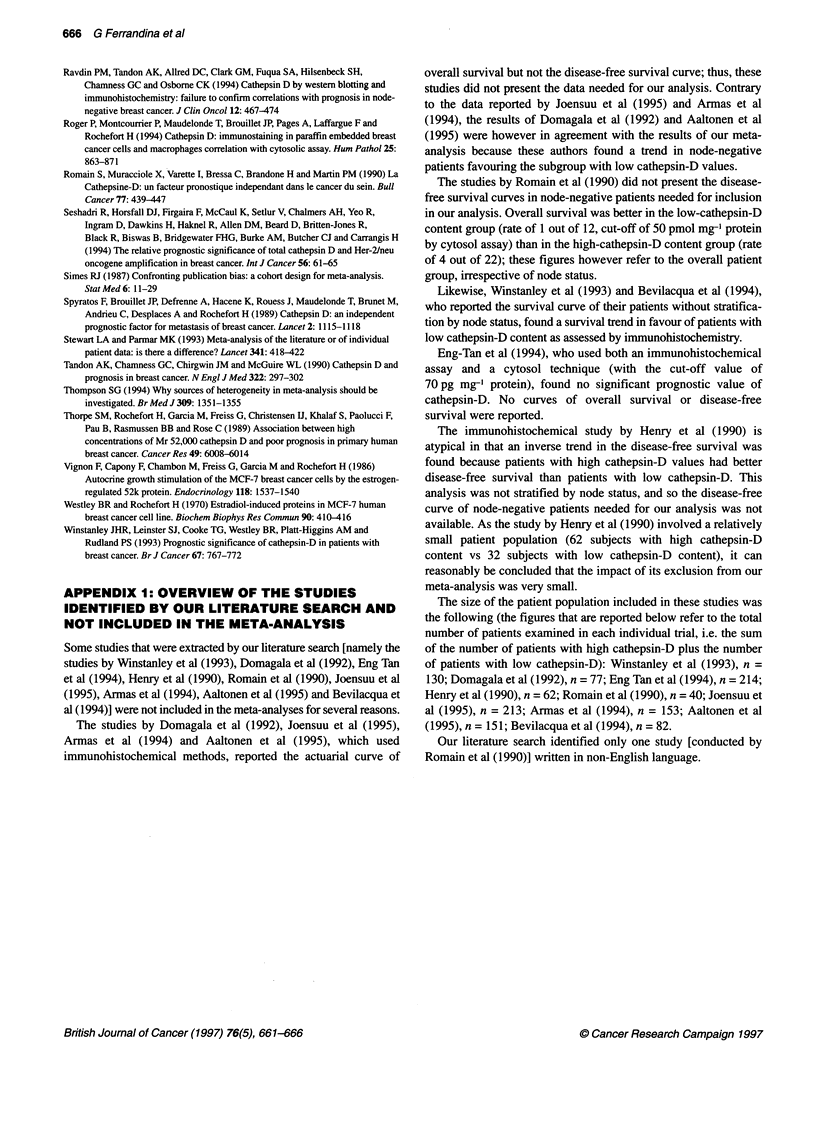# Relationship between cathepsin-D content and disease-free survival in node-negative breast cancer patients: a meta-analysis.

**DOI:** 10.1038/bjc.1997.442

**Published:** 1997

**Authors:** G. Ferrandina, G. Scambia, F. Bardelli, P. Benedetti Panici, S. Mancuso, A. Messori

**Affiliations:** Department of Gynaecology and Obstetrics, Catholic University, Rome, Italy.

## Abstract

Several reports have evaluated the correlation between cathepsin-D and overall survival or disease-free survival in node-negative breast cancer patients. Because conflicting data have so far been reported, a meta-analysis was conducted to clarify this problem. Eleven studies were included in our meta-analysis (total of 2690 patients). A specific meta-analytical methodology for censored data was used, and disease-free survival was the primary end point. Patients with low cathepsin-D levels had a significantly better disease-free survival than patients with high cathepsin-D values (meta-analytical odds ratio from 0.59 to 0.60 over the interval from 1 to 7 years). A secondary meta-analysis conducted exclusively on the data from eight studies based on cytosol assay gave substantially similar results. One limitation of our study is that the cut-off values to define high and low cathepsin-D concentrations were not identical in the various studies included in our meta-analysis (range from 20 to 78 pmol mg(-1) protein), thus introducing a possible bias in the statistical analysis of the data. However, a simulation based on the well-accepted method of the so-called publication bias showed that more than 100 null studies would be required to lead our results to a statistical level of non-significance. Considering the results of our meta-analysis, we conclude that the data presently available confirm a statistically significant association between high cathepsin-D values and poor disease-free survival in node-negative breast cancer patients.


British Journal of Cancer (1997) 76(5), 661–666
© 1997 Cancer Research Campaign

### Relationship between cathepsin-D content and disease-free survival in node-negative breast cancer patients: a meta-analysis

G Ferrandina[1], G Scambia[1], F Bardelli[2], P Benedetti Panici[1], S Mancuso[1], A Messori[2]

[1]Department of Gynaecology and Obstetrics, Catholic University, Rome, Italy; [2]Area SIFO di Metanalisi, Drug Information Centre, Policlinico di Careggi, Viale Morgagni 85, 50134 Florence, Italy

**Summary** Several reports have evaluated the correlation between cathepsin-D and overall survival or disease-free survival in node-negative breast cancer patients. Because conflicting data have so far been reported, a meta-analysis was conducted to clarify this problem. Eleven studies were included in our meta-analysis (total of 2690 patients). A specific meta-analytical methodology for censored data was used, and disease-free survival was the primary end point. Patients with low cathepsin-D levels had a significantly better disease-free survival than patients with high cathepsin-D values (meta-analytical odds ratio from 0.59 to 0.60 over the interval from 1 to 7 years). A secondary meta-analysis conducted exclusively on the data from eight studies based on cytosol assay gave substantially similar results. One limitation of our study is that the cut-off values to define high and low cathepsin-D concentrations were not identical in the various studies included in our meta-analysis (range from 20 to 78 pmol mg$^{-1}$ protein), thus introducing a possible bias in the statistical analysis of the data. However, a simulation based on the well-accepted method of the so-called publication bias showed that more than 100 null studies would be required to lead our results to a statistical level of non-significance. Considering the results of our meta-analysis, we conclude that the data presently available confirm a statistically significant association between high cathepsin-D values and poor disease-free survival in node-negative breast cancer patients.

**Keywords:** meta-analysis; cathepsin D; node-negative breast tumour

The identification of new prognostic factors, more closely related to tumour cell biology, would be of utmost importance for treatment planning in human breast cancer. Improvement in discrimination between low- and high-risk cases is of major concern, particularly in the subset of node-negative patients, 70% of whom are cured by surgery alone and would therefore be spared the cost and potential toxicity of adjuvant chemotherapy (McGuire, 1989; Copper, 1991). To date, several biological factors have been identified and proposed as potential prognostic indexes in human breast cancer. Among these, particular attention has been focused on proteolytic enzymes, such as cathepsin-D and urokinase-type plasminogen activator, which are involved in basement membrane/extracellular matrix degradation and tumour invasiveness and metastasis (Liotta et al, 1991).

Cathepsin-D, firstly identified as a 52-kDa oestrogen-regulated glycoprotein (Westley et al, 1970), displays both proteolytic activity in culture and an autocrine mitogenic activity in breast cancer cells (Vignon et al, 1986). The involvement of cathepsin-D in cancer invasion is also supported by the demonstration that transfection of cathepsin-D cDNA into rat tumorigenic cells increases their metastatic potential in nude mice (Garcia et al, 1990). In addition, higher cathepsin-D levels have been found in breast cancer patients with metastatic lymph node involvement than in node-negative patients (Pujol et al, 1993; Winstanley et al, 1993; Gion et al, 1995).

*Received 20 November 1996*
*Revised 12 March 1997*
*Accepted 12 March 1997*

*Correspondence to:* S Mancuso, Department of Gynecology and Obstetrics, Catholic University of the Sacred Heart, L.go Gemelli, 8, 00168 Rome, Italy

In recent years, great effort has been devoted to investigate the role of cathepsin-D as a possible marker of tumour invasiveness and poor prognosis, particularly in node-negative breast cancer patients. However, at present, the clinical usefulness of cathepsin-D measurement remains controversial; evidence has been reported that high cathepsin-D levels are associated with an unfavourable prognosis in node-negative breast cancer patients (Spyratos et al, 1989; Thorpe et al, 1989; Tandon et al, 1990; Kute et al, 1992; Isola et al, 1993), but some authors failed to find any relationship between cathepsin-D and clinical outcome (Namer et al, 1991; Kandalaft et al, 1993; Janicke et al, 1993; Pujol et al, 1993).

Inconsistency in the results may be due to variability of assay techniques, criteria of patient classification, different cut-off values of cathepsin-D assay and also to the low statistical power of individual studies that have often been conducted in relatively small patient series.

Meta-analysis provides an efficient tool for combining results of independent studies, thus increasing statistical power and possibly solving controversial issues.

In this report, we carried out a meta-analysis of the clinical studies evaluating the prognostic value of cathepsin-D in node-negative breast cancer patients.

### MATERIALS AND METHODS

### Literature search

We searched through the Iowa-IDIS compact disk database (Iowa Drug Information System, Iowa City, USA; computer search from January 1985 to September 1996) using 'cathepsin' and 'breast cancer' as index terms. This computer search was supplemented by consulting Current Contents (Current Contents on Diskette, Institute for Scientific Information, Philadelphia, USA; computer search of diskettes from October 1991 to September 1996), the Medline system on compact disk (Medline, Silver Platter International, Norwood, MA, USA; computer search of diskettes from January 1990 to September 1996), reviews, textbooks and experts in this particular field of study. Additionally, we reviewed all the references listed in the clinical studies that we found.

### Meta-analysis protocol

The criteria for inclusion of the clinical studies into our meta-analysis, as follows:

1. The clinical study regards patients with node-negative breast cancer in whom the levels of cathepsin-D were assayed at staging.
2. The study provides separate follow-up data for patients with 'high' or 'low' cathepsin-D values. The patient-specific end point of the follow-up is the occurrence of disease relapse.
3. 'High' or 'low' cathepsin-D values are defined according to cut-off values ranging from 20 to 78 pmol $mg^{-1}$ protein for cytosol assays or according to semiquantitative methods for immunohistochemical assays. Studies using cut-off values outside this range are excluded unless specific (staging and survival) data are available after patients' reclassification into two subgroups according to a cut-off value included in our accepted range.

Our meta-analysis of the disease-free survival data was conducted using an 'actuarial survival methodology' (see below) that requires the knowledge, for each study, of the number of events (or relapses) stratified for each time interval of the follow-up. As the information required by this type of meta-analysis corresponds to the availability of individual patient data with outcomes aggregated at follow-up intervals, an advantage of this meta-analytical methodology lies in its intermediate nature between meta-analyses of published data and meta-analyses of individual patient data (Stewart and Parmar, 1993).

### Statistical techniques

### *Survival meta-analysis*

This statistical method [which has previously been used by Gregory et al (1992), Messori et al (1994) and Berg et al (1994)] was described originally by Peto (1987). In our study, this type of meta-analysis was used to compare disease-free survival between patients with high cathepsin-D values and patients with low cathepsin-D values. All calculations were effected with a microcomputer program [program META.EXE (Messori and Rampazzo, 1993), Version 4.38]. According to Messori and Rampazzo (1993), the final result generated by the disease-free survival meta-analysis was denoted as 'log-rank odds-ratio' of meta-analysis.

Our primary meta-analysis of disease-free survival included the data of all clinical studies obtained from our literature search. Then, a secondary meta-analysis was conducted using exclusively the data of studies based on cytosol assays.

### *Other statistical calculations*

#### ***Extraction of raw survival data from the clinical studies***

In order to carry out our survival meta-analysis, the survival curves published in the various clinical studies were analysed by the method of Fine et al (1993). This method allows one to determine the distribution of the events and of the terminations of follow-up (i.e. cases of 'right-censored patients') stratified for each of the various time intervals of the follow-up. Controversial cases (in which this method provided time-specific survival rates, recomputed from raw data, that differed from the published actuarial curves) were solved by contacting the study's authors. The time intervals considered in this phase were the following: (1) from randomization to 12 months; (2) from 12 to 24 months; (3) from 24 to 36 months; (4) from 36 to 48 months; (5) from 48 to 60 months and (6) from 60 to 72 months.

**Table 1** Studies included in the meta-analysis

| Reference | No. of patients according to cathepsin-D content | | Cut-off (pmol $mg^{-1}$ protein) | Positivity (%) | Assay | Significance (*P*-value) |
|---|---|---|---|---|---|---|
| | Low | High | | | | |
| Isola et al (1993)[a] | 167 | 95 | | 36 | IHC[i] | 0.0001 |
| Janicke et al (1993) | 64 | 33 | 50 | 34 | ELSA | 0.077 |
| Kandalafi et al (1993)[b] | 84 | 51 | | 37.7 | IHC | 0.072 |
| Kute et al (1992) | 45 | 93 | 39 | 28 | RIA | 0.0001 |
| Namer et al (1991) | 132 | 114 | 35 | 46 | ELSA | NS |
| Pujol et al (1993) | 38 | 26 | 20 | 40 | ELISA | 0.07 |
| Seshadri et al (1994)[c] | 117 | 237 | 25 | 67 | ELSA | NS |
| Ravdin et al (1994) | 467 | 460 | 54 | 50 | Western blot, IHC | NS |
| Spyratos et al (1989) | 39 | 29 | 45 | 42.6 | ELISA | 0.001 |
| | 57 | 11 | 70 | 16 | ELSA | |
| Tandon et al (1990)[e] | 135 | 64 | 75 | 32 | Western blot | 0.0001 |
| Thorpe et al (1989)[f] | 93 | 26 | 78 | 22 | ELISA | 0.06 |
| Thorpe et al (1989)[g] | 24 | 57 | 24 | 70 | ELISA | 0.039 |

[a]At least 10% of strongly positive cells was used as cut-off value. [b]Authors used an H-score (0–2 = low, 3–5 = high cathepsin-D content) derived from the combination of a distribution score and an intensity score. [c]These data, directly provided by Seshadri and co-workers (1994) refer to the patient group whose follow-up information were updated to 31 May 1995. [d]These data were directly provided by the authors. Ravdin et al (1994) also used an immunohistochemical assay the results of which have not been considered herein. [e]The cut-off used is 75 absorbance units. [f]Subgroup of premenopausal patients. [g]subgroup of post-menopausal patients. [h]Percentage of patients with high cathepsin-D values. [i]IHC, immunohistochemistry.

 © Cancer Research Campaign 1997

***Calculation of pooled rates*** In the survival meta-analysis, the pooled disease-free survival rates for the high cathepsin-D group were estimated from the raw data using non-meta-analytical actuarial methods (i.e. actuarial analysis of crude survival data stratified by time interval and summed over all studies). The pooled rates for low cathepsin group [with 95% confidence intervals (CIs)] were computed by the method of Laupacis et al (1988).

***Assessment of the inter-study heterogeneity*** There is a growing agreement about the need to perform a heterogeneity assessment in all meta-analyses (Thompson, 1994). The inter-study heterogeneity was estimated using the equations reported in the appendix of Collins et al (1985) and in Section 2.2.2 of Messori and Rampazzo (1993).

***Publication bias calculations*** The issue of publication bias (Simes, 1987) was addressed by the procedure of Rosenthal (Klein et al, 1986), which is based on the estimation of the minimum number *m* of negative (or null) studies required to lead a significant meta-analysis to non-significance. The value of *m* was calculated by the formula described by Klein et al (1986). The *m* negative (or null) studies are hypothetical (simulated) trials in which the two groups being compared are supposed to be identical in terms of outcome parameters. A highly significant meta-analysis can be reversed to non-significance only by large values of *m* and vice versa.

### RESULTS

### Literature search

Our literature search identified a total of 11 controlled clinical studies that met the inclusion criteria of our meta-analysis (Table 1). As regards the studies by Pujol et al (1993), by Seshadri et al (1994) and by Ravdin et al (1994), we obtained the disease-free survival data of node-negative patients directly from the authors because these data were not explicitly reported in the published articles. The Appendix summarizes the characteristics of the studies that were identified by our literature search but did not meet the inclusion criteria of the meta-analysis.

### Meta-analysis including all studies

The results of our meta-analysis of disease-free survival are shown in Table 2. The relative risk of relapse (expressed as odds ratios) was significantly different between patients with high vs low cathepsin-D values.

In our primary meta-analysis, the assessment of inter-study heterogeneity gave a chi-square of 91.6 (d.f. = 11, $P = 0.001$) at 84 months. These data show that the inter-study heterogeneity was remarkably high.

The publication bias calculations indicated that the number of null studies needed to lead the meta-analysis results to levels of statistical non-significance was equal to 111 (estimate based on the odds ratio at 60 months).

**Table 2** Study-specific survival rates at 12, 24, 36, 48, 60, 72 and 84 months[a] and pooled rates generated by the survival meta-analysis according to cathepsin-D status

| | Disease-free survival rates (%)[a] | | | | | | | | | | | | | |
|---|---|---|---|---|---|---|---|---|---|---|---|---|---|---|
| | At 12 months | | At 24 months | | At 36 months | | At 48 months | | At 60 months | | At 72 months | | At 84 months | |
| | Low[b] | High[b] | Low | High | Low | High | Low | High | Low | High | Low | High | Low | High |
| Isola et al (1993) | 92 | 85 | 87 | 74 | 81 | 60 | 80 | 54 | 74 | 49 | 69 | 49 | 69 | 47 |
| Janicke et al (1993) | 97 | 94 | 95 | 82 | 92 | 73 | 92 | 73 | 92 | 73 | NA | NA | NA | NA |
| Kandalaft et al (1993) | 99 | 96 | 92 | 86 | 90 | 80 | 77 | 73 | 77 | 65 | 77 | 65 | 77 | 65 |
| Kute et al (1992) | 97 | 93 | 97 | 88 | 97 | 83 | 93 | 76 | 88 | 71 | 88 | 66 | 88 | 53 |
| Namer et al (1991) | 97 | 100 | 93 | 97 | 88 | 93 | 88 | 93 | 84 | 87 | 84 | 88 | 84 | 88 |
| Pujol et al (1993) | 100 | 92 | 97 | 84 | 94 | 76 | 92 | 76 | 92 | 76 | 92 | 76 | NA | NA |
| Ravdin et al (1994) | 95 | 96 | 90 | 89 | 84 | 80 | 82 | 77 | 78 | 73 | 75 | 70 | 71 | 70 |
| Seshadri et al (1994) | 98 | 96 | 96 | 88 | 89 | 83 | 84 | 82 | 81 | 79 | 78 | 78 | 60 | 78 |
| Spyratos et al (1989) | 100 | 96 | 97 | 96 | 94 | 79 | 94 | 68 | 94 | 62 | 81 | 31 | 81 | 31 |
| Tandon et al (1990) | 96 | 83 | 90 | 71 | 81 | 57 | 78 | 53 | 71 | 47 | 71 | 47 | NA | NA |
| Thorpe et al (1989)[c] | 90 | 81 | 82 | 65 | 77 | 53 | 69 | 45 | 69 | 45 | 69 | 45 | 69 | 45 |
| Thorpe et al (1989)[d] | 96 | 89 | 96 | 83 | 91 | 77 | 86 | 73 | 86 | 69 | 85 | 69 | 85 | 69 |
| Pooled rates (%) | 97.4 | 94.0 | 95.6 | 86.4 | 90.2 | 78.6 | 86.8 | 75.1 | 83.2 | 71.1 | 80.8 | 69.1 | 79.5 | 68.1 |
| 95% CI | (94.7–100) | | (91.5–100) | | (85.8–94.8) | | (82.6–91.3) | | (78.9–87.5) | | (76.6–85.1) | | (75.3–83.7) | |
| Relative risk of relapse[e] | 0.62 | 1 | 0.53 | 1 | 0.55 | 1 | 0.57 | 1 | 0.59 | 1 | 0.60 | 1 | 0.61 | 1 |
| (95% CI) | (0.43–0.89) | – | (0.41–0.69) | – | (0.45–0.69) | – | (0.47–0.69) | – | (0.49–0.70) | – | (0.51–0.72) | – | (0.52–0.73) | – |
| $z$[f] | 2.58 | | 4.87 | | 5.67 | | 5.77 | | 5.89 | | 5.74 | | 5.56 | |
| Statistical significance[f] | $P = 0.01$ | | $P < 0.001$ | | $P < 0.001$ | | $P < 0.001$ | | $P < 0.001$ | | $P < 0.001$ | | $P < 0.001$ | |

[a]To ensure the homogeneity of calculations, these study-specific rates were all recomputed from the survival data generated by the application of Fine's method (1993). This recomputation was made using Equation 4b of Kaplan and Meier (1958). [b]'Low' and 'High' refer to cathepsin-D content. [c]Subgroup of premenopausal patients. [d]Subgroup of post-menopausal patients. [e]The relative risk, estimated as log-rank odds ratios and the statistical comparisons refer to the whole period from time zero to the individual timepoint. [f]These values refer to the meta-analytical comparisons between the high cathepsin-D and the low Cathepsin-D groups made at the various time-points of the follow-up.

This assessment of publication bias tested the hypothesis of a greater likelihood of positive studies being published, but obviously could not check the stability of our results against the likelihood that the results of some studies might have been inflated by the use of an optimum cut-off point.

The data of Spyratos et al (1989) were introduced in our meta-analysis using the cut-off of 45 pmol $mg^{-1}$. In a separate analysis (data not shown), we checked that the results of our meta-analysis remain virtually unchanged using the cut-off of 70 pmol $mg^{-1}$ reported by Spyratos et al (1989).

### Meta-analysis including cytosol-based studies

The eight papers by Pujol et al (1993), Spyratos et al (1989), Kute et al (1992), Namer et al (1991), Janicke et al (1993), Tandon et al (1990), Seshadri et al (1994) and Ravdin et al (1994) (Table 1) were included in this secondary meta-analysis focused on studies using cytosol assays. The results were very similar to those produced by the first meta-analysis. Statistical significance was slightly less marked (value at 24 months: $z = 3.96$, $P < 0.001$; value at 48 months: $z = 4.36$, $P < 0.001$; value at 72 months: $z = 4.61$, $P < 0.001$); the values of odds ratios at the various times were all around 0.60 (value at 24 months: 0.56 with 95% CI of 0.42–0.75; value at 48 months: 0.62 with 95% CI of 0.50–0.77; value at 72 months: 0.63 with 95% CI of 0.52–0.77). Interestingly enough, the level of inter-study heterogeneity showed no decrease after the exclusion of studies using immunohistochemical techniques.

### DISCUSSION

To our knowledge, this is the first study in which the possible prognostic role of a biological factor has been evaluated by means of a meta-analytical approach based on survival methodology. One advantage of the meta-analytical approach is that it enables the circumvention of the lack of statistical power as a result of the relatively small sample size of many studies. The choice to carry out this meta-analysis on the prognostic role of cathepsin-D in node-negative breast cancer patients stems from the following reasons: (1) evidence has been reported about the direct relationship of cathepsin-D with tumour cell invasiveness and metastatic behaviour (Garcia et al, 1990; Pujol et al, 1993; Winstanley et al, 1993; Gion et al, 1995); (2) cathepsin-D has been included among the biological factors potentially useful for discrimination between high- and low-risk patients to avoid adjuvant overtreatment in the latter group (Bevilacqua et al, 1994); (3) although the negative prognostic role of high cathepsin-D levels in node-negative breast cancer patients has been demonstrated by several authors (Spyratos et al, 1989; Thorpe et al, 1989; Tandon et al, 1990; Kute et al, 1992; Isola et al, 1993), conflicting results have also been reported (Namer et al, 1991; Kandalaft et al, 1993; Janicke et al, 1993; Pujol et al, 1993). Our study demonstrated that elevated cathepsin-D values identify node-negative breast cancer patients characterized by unfavourable prognosis in terms of disease-free survival. Although heterogeneity of the studies as well as different definitions of cathepsin-D positivity might have been a source of bias, the exclusion of studies analysing cathepsin-D content by immunohistochemistry did not change the statistical significance of our meta-analytical results. Moreover, despite a certain degree of heterogeneity in the percentage of cathepsin-D positivity, our publication bias simulations demonstrated that more than 100 null studies would be required to reverse our results to the level of statistical non-significance.

It should be noted that several reports could not be included in our meta-analysis because of the incomplete presentation of survival curves. This fact emphasizes the need for studies dealing with the assessment of potentially prognostic biological factors to ensure a sufficient level of reporting of the results to allow reappraisal in meta-analysis studies.

It has been suggested that the relationship of total tumour cytosolic cathepsin-D to adverse prognosis may be impaired by the presence of cathepsin-D in non-epithelial cells. In particular, it has been found that stromal and macrophage-like cells are cathepsin-D positively immunostained in approximately 35% of cases defined as negative according to tumour cell immunoreactions irrespective of the use of monoclonal (Isola et al, 1993) or polyclonal (Domagala et al, 1992) antibodies. However, Roger et al (1994) reported that cytosolic cathepsin-D levels correlated with cathepsin-D expression in cancer cells, and several studies agree with the finding that cathepsin-D contents in stromal and cancer cells are directly correlated (Isola et al, 1993; Eng Tan et al, 1994; Ravdin et al, 1994).

On the other hand, results obtained by immunohistochemistry showed the highest degree of heterogeneity, probably because of differences in the antibodies used and in cathepsin D positivity criteria (see Table 2); some studies demonstrated the adverse prognostic role of tumour cell cathepsin-D content (Isola et al, 1993; Winstanley et al, 1993; Roger et al, 1994), while others failed to find any relationship between tumour cell cathepsin-D expression and clinical outcome (Domagala et al, 1992; Kandalaft et al, 1993; Armas et al, 1994) and only one (Henry et al, 1990) showed a favourable impact of tumour cell cathepsin-D on prognosis. Ravdin et al (1994) suggested that cathepsin-D assessment by Western blot should not be routinely used for the prognostic characterization of breast cancer patients.

Although a general consensus on the routine use of this technique is still far from being achieved, the assessment of cathepsin-D content by means of immunoradiometric assay seems to be likely to give more reliable and comparable inter-study results as assessed by the EORTC Receptor Study Group (Benraad et al, 1992).

One limitation of our study is that the cut-off values that differentiate between high and low cathepsin-D concentrations were not identical in the various studies but varied from 20 to 78 pmol $mg^{-1}$ protein. While this fact could have introduced a bias increasing the statistical significance of our results, the high number of null studies required in our publication bias assessment to reverse our results to non-significance supports the conclusion of our analysis.

We cannot rule out the possibility that some of the studies included in our analysis might have been influenced by the selection of an optimum cut-off point, but the overall evidence emerging from our study seems to be sufficient to support the conclusion that cathepsin-D has a prognostic role in these patients.

In their paper that suggested a poor correlation between prognosis and cathepsin-D levels, Ravdin et al (1994) conducted an exploratory analysis on different cathepsin-D cut-off values wherein these cut-off values were retrospectively varied over the range from 1 to 1000 units. This analysis showed that the 'optimum' cut-off value (i.e. the value of Cathepsin-D that produced the highest statistical correlation with a $P$-value of 0.009) was around 22 units. Furthermore, Ravdin et al (1994) tried to ascertain to what extent this retrospective identification of the optimum cut-off could have contributed to an artifactual $P$-value

*An analytical printout of the survival data of the clinical studies included in our meta-analysis is available from the authors upon request.

© Cancer Research Campaign 1997

(i.e. to an overestimation of the statistical significance of the correlation between cathepsin-D levels and prognosis) and to what extent their findings, which revealed this apparent correlation using the retrospective cut-off of 22 units, could be compatible with a purely casual result (i.e. a result derived from a simulated population wherein the correlation was totally absent). This latter analysis of Ravdin et al (1994), which was based on the simulation of about 300 different data sets, showed that there was a one in six chance that the high correlation found in the primary analysis was purely casual. The authors therefore concluded that their hypothesis of a 'true' correlation was not supported by sufficient evidence and that the apparent cut-off point found retrospectively in their real data set was likely to be casual.

While these conclusions proposed by Ravdin et al (1994), after analysis of their data, are perhaps too conservative, it should be stressed that the findings reported by these authors are however consistent with a correlation at *P*-levels of about 0.10 or 0.20 and therefore suggest at least the presence of a statistical trend.

To better interpret the results of our meta-analysis, we tried to plan a simulation based on the comparison of a hypothetical population with no correlation vs our real patient population. Unfortunately, the lack of individual patient data on cathepsin D assays did not allow us to produce reliable statistical results on this point.

Another controversial point regards the methodology of the survival meta-analysis used in our study. Because there is presently no consensus on which methodology should be recommended for survival meta-analysis, we analysed all survival data included in our study using a different method of survival meta-analysis (Simes, 1987) in which median survival is used for comparing two patient groups with one another across a series of different clinical studies. This further analysis (data not shown) produced essentially the same results obtained by our primary survival meta-analysis.

Therefore considering the results of our primary meta-analysis (together with the relatively low inter-study difference in the cut-off values and the results of our publication bias assessments), we conclude that the data presently available confirm a statistically significant association between cathepsin-D and disease-free survival in breast cancer.

### ACKNOWLEDGEMENTS

We greatly acknowledge Dr Pascal Pujol and Dr Henri Rochefort (Montpellier, France), Dr Ram Seshadri and Dr Kieran McCaul (Bedford Park, Australia), Dr Peter M Ravdin and Dr Gary M Clark (San Antonio, USA) for their help in providing patient survival data. This study was supported by the Italian Association for Cancer Research (AIRC).

### REFERENCES

Aaltonen M, Lipponen P, Kosma VM, Aaltomaa S and Syrjanen K (1995) Prognostic value of Cathepsin-D expression in female breast cancer. *Anticancer Res* **15**: 1033–1037

Armas OA, Gerald WL, Lesser ML, Arroyo CA, Norton ML and Rosen PP (1994) Immunohistochemical detection of Cathepsin D in $T_2 N_0 M_0$ breast carcinoma. *Am J Surg Pathol* **18**: 158–166

Berg AT and Shinnar S (1994) Relapse following discontinuation of antiepileptic drugs: a meta-analysis. *Neurology* **44**: 601–608

Benraad TJ, Geurtsmoespot A, Sala M, Piffanelli A, Ross A and Foekens JA on Behalf of the EORTC Receptor Study Group (1992) Quality control of Cathepsin D measurement by the EORTC receptor study group. *Eur J Cancer* **28**: 72–75

Bevilacqua P, Boracchi P and Gasparini G (1994) Prognostic indicators for early-stage breast carcinoma. Part II; value of Cathepsin D expression, detected by immunocytochemistry. A multiparametric study. *Int J Oncol* **5**: 559–565

Collins R, Yusuf S and Peto R (1985) Overview of randomised trials of diuretics in pregnancy. *Br Med J* **290**: 17–25

Copper MR (1991) The role of chemotherapy for node-negative breast cancer. *Cancer* **67**: 1744–1747

Domagala W, Striker G, Weber A and Osborne M (1992) Cathepsin D in invasive ductal NOS breast carcinoma as defined by immunohistochemistry. No correlation with survival at 5 years. *Am J Pathol* **141**: 1003–1012

Eng Tan P, Benz CC, Dollbaum C, Moore II DH, Edgerton SM, Zawa DT and Thor AD (1994) Prognostic value of Cathepsin D expression in breast cancer: immunohistochemical assessment and correlation with radiometric assay. *Ann Oncol* **5**: 329–336

Fine HA, Dear KBG, Loeffler JS, Black PML and Canellos GP (1993) Meta-analysis of radiation therapy with and without adjuvant chemotherapy for malignant gliomas in adults. *Cancer* **71**: 2585–2592

Garcia D, Derocq D, Pujol P and Rochefort H (1990) Overexpression of transfected cathepsin D in transformed cells increases their malignant phenotype and metastatic potency. *Oncogene* **5**: 1809–1814

Gion M, Mione R, Dittadi R, Romanelli M, Pappagallo L, Capitanio G, Friede U, Barbazza R, Visiona A and Dante S (1995) Relationship between cathepsin D and other pathological and biological parameters in 1752 patients with primary breast cancer. *Eur J Cancer* **31**: 671–677

Gregory WM, Richards MA and Malpas JS (1992) Combination chemotherapy versus melphalan and prednisolone in the treatment of multiple myeloma: an overview of published trials. *J Clin Oncol* **10**: 334–340

Henry JA, McCarthy AL, Angus B, Westley BR, May FEB, Nicholson S, Cairns J, Harris AL and Horne CHW (1990) Prognostic significance of the estrogen-regulated protein, cathepsin D, in breast cancer. An immunohistochemical study. *Cancer* **65**: 265–271

Isola J, Weitz S, Visakorpi T, Holli K, Shea R, Knabbaz N and Kallioniemi OP (1993) Cathepsin D expression detected by immunohistochemistry has independent value in axillary node-negative breast cancer. *J Clin Oncol* **11**: 36–43

Janicke F, Schmitt M, Pache L, Ulm K, Harbeck N, Hofler H and Graeff H (1993) Urokinase (uPA) and its inhibitor PAI-1 are strong and independent prognostic factors in node-negative breast cancer. *Breast Cancer Res Treat* **24**: 195–208

Joensuu H, Toikkanen S and Isola J (1995) Stromal cell Cathepsin D expression and long-term survival in breast cancer. *Br J Cancer* **71**: 155–159

Kandalaft PK, Chang KL, Ahn CW, Traweek ST, Mehta P and Battifora H (1993) Prognostic significance of immunohistochemical analysis of cathepsin D in low-stage breast cancer. *Cancer* **71**: 2756–2763

Kaplan EL and Meier P (1958) Nonparametric estimation from incomplete observations. *Am Statist Assoc J* **53**: 457–483

Klein S, Simes J and Blackburn GL (1986) Total parenteral nutrition and cancer clinical trials. *Cancer* **58**: 1378–1386

Kute TE, Shao ZM, Sugg NK, Long RT, Russel GB and Case D (1992) Cathepsin D as a prognostic indicator for node-negative breast cancer patients using immunoassays and enzymatic assays. *Cancer Res* **52**: 5198–5203

Laupacis A, Sackett DL and Roberts RS (1988) An assessment of clinically useful measures of the consequences of treatment. *N Engl J Med* **318**: 1728–1733

Liotta LA, Stetler-Stevenson WG and Steeg PS (1991) Cancer invasion and metastasis: positive and negative regulatory elements. *Cancer Invest* **9**: 543–551

McGuire WL (1989) Adjuvant therapy of node-negative breast cancer. *N Engl J Med* **320**: 525–527

Messori A and Rampazzo R (1993) Meta-analysis of clinical trials based on censored end-points: simplified theory and implementation of the statistical algorithms on a microcomputer. *Comput Progr Meth Biomed* **40**: 261–267

Messori A, Brignola C, Trallori G, Rampazzo R, Bardazzi G, Belloli C, D'Albasio G, De Simone G and Martini N (1994) Effectiveness of 5-aminosalicylic acid for maintaining remission in patients with Crohn's disease: a meta-analysis. *Am J Gastroenterol* **89**: 692–698

Namer M, Ramaioli A, Fontana X, Etienne MC, Hry M, Jourlait A, Milano G, Frenay M, Francois E and Lapalus F (1991) Prognostic value of total cathepsin D in breast tumors. *Breast Cancer Res Treat* **19**: 85–93

Peto R (1987) Why do we need systematic overviews of randomised trials. *Stat Med* **6**: 233–239

Pujol P, Maudelonde T, Daures JP, Rouanet P, Brouillet JP, Pujol H and Rochefort H (1993) A prospective study of the prognostic value of cathepsin D levels in breast cancer cytosol. *Cancer* **71**: 2006–2012

© Cancer Research Campaign 1997 

Ravdin PM, Tandon AK, Allred DC, Clark GM, Fuqua SA, Hilsenbeck SH, Chamness GC and Osborne CK (1994) Cathepsin D by western blotting and immunohistochemistry: failure to confirm correlations with prognosis in node-negative breast cancer. *J Clin Oncol* **12**: 467–474

Roger P, Montcourrier P, Maudelonde T, Brouillet JP, Pages A, Laffargue F and Rochefort H (1994) Cathepsin D: immunostaining in paraffin embedded breast cancer cells and macrophages correlation with cytosolic assay. *Hum Pathol* **25**: 863–871

Romain S, Muracciole X, Varette I, Bressa C, Brandone H and Martin PM (1990) La Cathepsine-D: un facteur pronostique independant dans le cancer du sein. *Bull Cancer* **77**: 439–447

Seshadri R, Horsfall DJ, Firgaira F, McCaul K, Setlur V, Chalmers AH, Yeo R, Ingram D, Dawkins H, Haknel R, Allen DM, Beard D, Britten-Jones R, Black R, Biswas B, Bridgewater FHG, Burke AM, Butcher CJ and Carrangis H (1994) The relative prognostic significance of total cathepsin D and Her-2/neu oncogene amplification in breast cancer. *Int J Cancer* **56**: 61–65

Simes RJ (1987) Confronting publication bias: a cohort design for meta-analysis. *Stat Med* **6**: 11–29

Spyratos F, Brouillet JP, Defrenne A, Hacene K, Rouess J, Maudelonde T, Brunet M, Andrieu C, Desplaces A and Rochefort H (1989) Cathepsin D: an independent prognostic factor for metastasis of breast cancer. *Lancet* **2**: 1115–1118

Stewart LA and Parmar MK (1993) Meta-analysis of the literature or of individual patient data: is there a difference? *Lancet* **341**: 418–422

Tandon AK, Chamness GC, Chirgwin JM and McGuire WL (1990) Cathepsin D and prognosis in breast cancer. *N Engl J Med* **322**: 297–302

Thompson SG (1994) Why sources of heterogeneity in meta-analysis should be investigated. *Br Med J* **309**: 1351–1355

Thorpe SM, Rochefort H, Garcia M, Freiss G, Christensen IJ, Khalaf S, Paolucci F, Pau B, Rasmussen BB and Rose C (1989) Association between high concentrations of Mr 52,000 cathepsin D and poor prognosis in primary human breast cancer. *Cancer Res* **49**: 6008–6014

Vignon F, Capony F, Chambon M, Freiss G, Garcia M and Rochefort H (1986) Autocrine growth stimulation of the MCF-7 breast cancer cells by the estrogen-regulated 52k protein. *Endocrinology* **118**: 1537–1540

Westley BR and Rochefort H (1970) Estradiol-induced proteins in MCF-7 human breast cancer cell line. *Biochem Biophys Res Commun* **90**: 410–416

Winstanley JHR, Leinster SJ, Cooke TG, Westley BR, Platt-Higgins AM and Rudland PS (1993) Prognostic significance of cathepsin-D in patients with breast cancer. *Br J Cancer* **67**: 767–772

### APPENDIX 1: OVERVIEW OF THE STUDIES IDENTIFIED BY OUR LITERATURE SEARCH AND NOT INCLUDED IN THE META-ANALYSIS

Some studies that were extracted by our literature search [namely the studies by Winstanley et al (1993), Domagala et al (1992), Eng Tan et al (1994), Henry et al (1990), Romain et al (1990), Joensuu et al (1995), Armas et al (1994), Aaltonen et al (1995) and Bevilacqua et al (1994)] were not included in the meta-analyses for several reasons.

The studies by Domagala et al (1992), Joensuu et al (1995), Armas et al (1994) and Aaltonen et al (1995), which used immunohistochemical methods, reported the actuarial curve of overall survival but not the disease-free survival curve; thus, these studies did not present the data needed for our analysis. Contrary to the data reported by Joensuu et al (1995) and Armas et al (1994), the results of Domagala et al (1992) and Aaltonen et al (1995) were however in agreement with the results of our meta-analysis because these authors found a trend in node-negative patients favouring the subgroup with low cathepsin-D values.

The studies by Romain et al (1990) did not present the disease-free survival curves in node-negative patients needed for inclusion in our analysis. Overall survival was better in the low-cathepsin-D content group (rate of 1 out of 12, cut-off of 50 pmol $mg^{-1}$ protein by cytosol assay) than in the high-cathepsin-D content group (rate of 4 out of 22); these figures however refer to the overall patient group, irrespective of node status.

Likewise, Winstanley et al (1993) and Bevilacqua et al (1994), who reported the survival curve of their patients without stratification by node status, found a survival trend in favour of patients with low cathepsin-D content as assessed by immunohistochemistry.

Eng-Tan et al (1994), who used both an immunohistochemical assay and a cytosol technique (with the cut-off value of 70 pg $mg^{-1}$ protein), found no significant prognostic value of cathepsin-D. No curves of overall survival or disease-free survival were reported.

The immunohistochemical study by Henry et al (1990) is atypical in that an inverse trend in the disease-free survival was found because patients with high cathepsin-D values had better disease-free survival than patients with low cathepsin-D. This analysis was not stratified by node status, and so the disease-free curve of node-negative patients needed for our analysis was not available. As the study by Henry et al (1990) involved a relatively small patient population (62 subjects with high cathepsin-D content vs 32 subjects with low cathepsin-D content), it can reasonably be concluded that the impact of its exclusion from our meta-analysis was very small.

The size of the patient population included in these studies was the following (the figures that are reported below refer to the total number of patients examined in each individual trial, i.e. the sum of the number of patients with high cathepsin-D plus the number of patients with low cathepsin-D): Winstanley et al (1993), $n$ = 130; Domagala et al (1992), $n$ = 77; Eng Tan et al (1994), $n$ = 214; Henry et al (1990), $n$ = 62; Romain et al (1990), $n$ = 40; Joensuu et al (1995), $n$ = 213; Armas et al (1994), $n$ = 153; Aaltonen et al (1995), $n$ = 151; Bevilacqua et al (1994), $n$ = 82.

Our literature search identified only one study [conducted by Romain et al (1990)] written in non-English language.

© Cancer Research Campaign 1997